# Clinical, neuroimaging, and nerve conduction characteristics of spontaneous Conus Medullaris infarction

**DOI:** 10.1186/s12883-019-1566-1

**Published:** 2019-12-17

**Authors:** Yi-Ching Weng, Shy-Chyi Chin, Yah-Yuan Wu, Hung-Chou Kuo

**Affiliations:** 1 Department of Neurology, Chang Gung Memorial Hospital at Linkou Medical Center, Taoyuan, Taiwan; 2 Medical Imaging and Intervention, Chang Gung Memorial Hospital at Linkou Medical Center, Taoyuan, Taiwan; 3 Chang Gung University College of Medicine, Taoyuan, Taiwan

**Keywords:** Conus medullaris infarction, Spinal cord infarction, Cauda equina syndrome, Electromyography

## Abstract

**Background:**

Spontaneous conus medullaris infarction is a rare disease. We describe two patients with spontaneous conus medullaris infarction presenting as acute cauda equina syndrome and their unique electromyography (EMG) findings.

**Case presentation:**

Two patients developed acute low back pain with mild asymmetric paraparesis, loss of perianal sensation and sphincter dysfunction. Ankle deep tendon reflexes were reduced in bilaterally. Neither patient had cardiovascular risk factors. Magnetic Resonance imaging showed infarction in the conus medullaris. Functional recovery was good in both patients, but progressive asymmetric calf wasting and sphincter dysfunction remained. EMG studies at follow-up of at least 3 years demonstrate active denervation at the muscles innervated by the first sacrum anterior horn cells.

**Conclusion:**

Spontaneous conus medullaris infarction can occur in healthy individuals and presents as cauda equina syndrome. Findings of needle EMG studies indicate a progressive course of sacrum anterior horn cell disorder during long-term follow-up.

## Background

Spinal cord infarction (SCI) is rare, accounting for about 1% of stroke and 6–8% of acute myelopathy [1, 2]. This lesion is most common in the thoracolumbar region, followed by the mid-cervical area [2, 3]. The initial presentation of SCI is the sudden or acute onset of back pain associated with motor, sensory, and autonomic system dysfunction. Diagnosis is based on clinical presentations and magnetic resonance imaging (MRI) of the spine. Differential diagnosis includes cord compression lesions such as herniated disc, epidural hematoma, tumor, and spinal canal stenosis. Transverse myelitis, multiple sclerosis, and vascular deformity should also be considered. Infarction in the conus medullaris is particularly rare because this region has a relatively sufficient blood. Herein, we report 2 patients with conus medullaris infarction presenting as cauda equina syndrome. We review their clinical manifestations and spinal images. In addition, we present nerve conduction studies and electromyography (EMG) for both patients, assessments that are rarely performed in patients with conus medullaris infarction. The potential pathological mechanism underlying the unique EMG presentations are discussed.

## Case presentation

Two patients with conus medullaris infarction were admitted to the neurology ward at a medical center Chang Gung Memorial Hospital in Taiwan. The protocol was approved by the ethics committee of Chang Gung Memorial Hospital (IRB No.: 201600290B0). The study conforms to the Declaration of Helsinki as revised in 2013, and written informed consent for study publication was obtained from our two participants.

To review the literature, we used the PubMed data base to search the terms ‘spinal cord infarction,’ and ‘MRI,’ and ‘electromyography.’ We searched for case reports of conus medullaris infarction and cauda equina syndrome published in the English language with data reported at the individual level.

### Patient 1

A 55-year-old male with dyslipidemia and no history of hypertension, diabetes mellitus, smoking, alcohol consumption, or special family history was admitted for paresis of the lower limbs. He experienced the sudden onset of sharp pain in the lower back upon rising after lying on the sofa for about 30 min. He then experienced bilateral leg weakness that was worse on the left side, urinary incontinence, constipation, and numbness in the sacrum. He also experienced erectile dysfunction the next morning. Initial neurologic examinations were performed 20 h after symptom onset and confirmed bilateral lower limb weakness. The Medical Research Council Scale for Muscle Strength score was 4 out of 5 in both thighs and right foot and less than 3 out of 5 in the left foot. The deep tendon reflex was hyporeflexive in both knees and absent in both ankles. In both feet, the Babinski sign was observed upon stimulation of the plantar reflex. Examination of sensory modalities revealed saddle dysesthesia, asymmetric hyperesthesia on both L5 and S1 dermatomes, and impaired position sense in both feet. The clinical manifestations were similar to those of cauda equina syndrome. His muscle strength gradually improved after 5 days, with remaining weakness in the left calf muscles. Asymmetric atrophy of the calf muscles was noted 3 years later. His neurologic sequel included difficulty walking on toes on the left side, mild perianal dysesthesia, and neurogenic bladder.

Blood biochemical analysis conducted 32 h after symptom onset revealed normal concentrations of electrolytes, liver enzymes, blood urea nitrogen, creatinine, and triglycerides and elevated levels of total cholesterol (237 mg/dL; normal range, < 200 mg/dL) and low-density lipoprotein (178 mg/dL; normal range, < 130 mg/dL). Routine blood tests showed normal values for hemoglobin, hematocrit, leukocytes, platelets, prothrombin time, partial thromboplastin time, and erythrocyte sedimentation rate. The plasma levels of fibrinogen, D-dimer, protein C, protein S, antithrombin III, homocysteine, and anticardiolipin antibody were all within normal limits. The serological tests for human immunodeficiency virus infection, venereal disease, lupus anticoagulant, and serum antinuclear antibody were negative. Cerebrospinal fluid examination 42 h after symptom onset showed normal cell counts and glucose level and a mildly elevated protein level (49 mg/dL; normal range, 15–45 mg/dL). The electrocardiogram (ECG) revealed sinus bradycardia. The Holter ECG monitor report was normal. Transthoracic echocardiogram revealed no intra-cardiac thrombus.

**Fig. 1:**
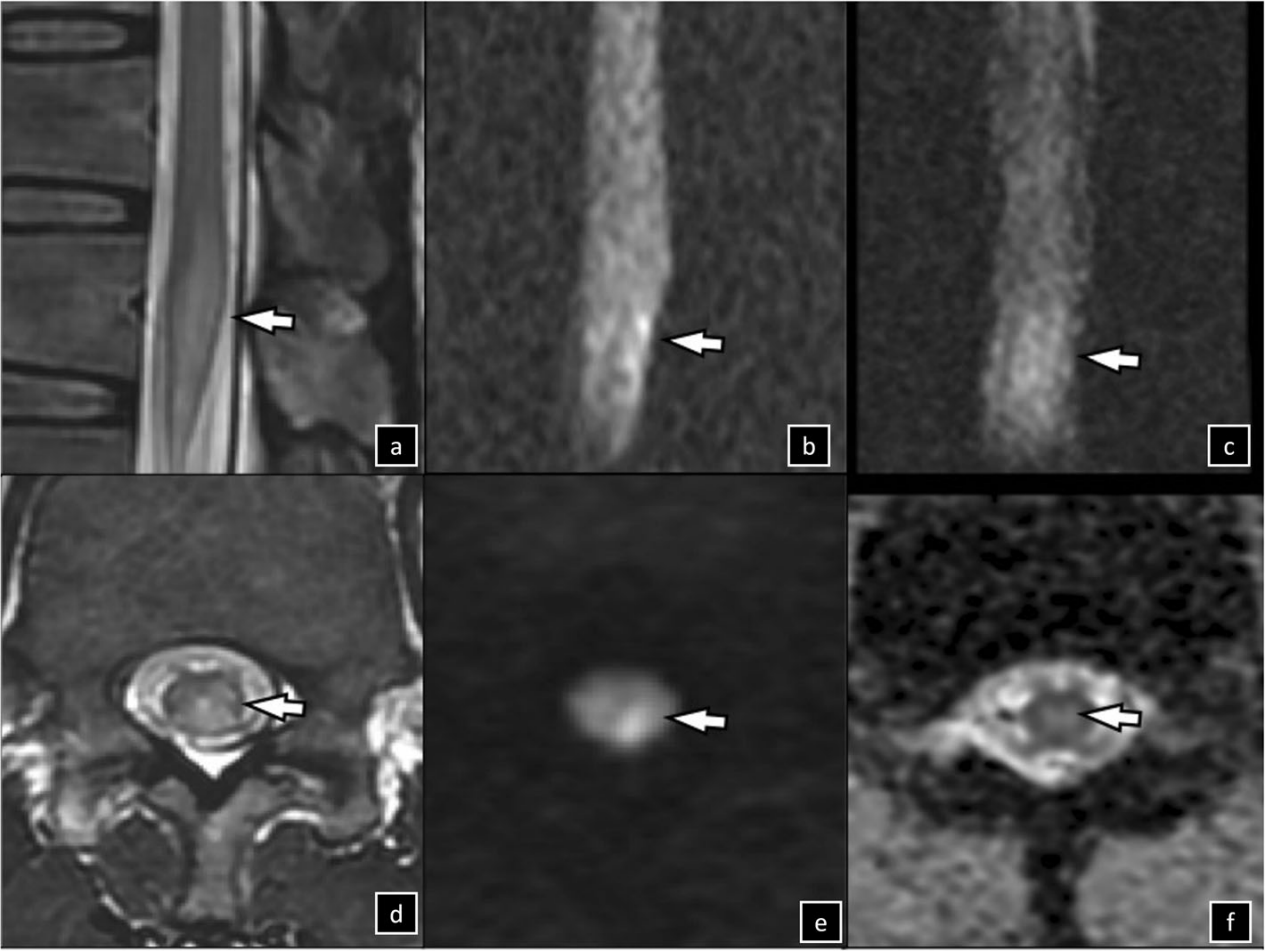
Spine MRI of patient 1, 9 days after symptoms onset. High signal intensity of sagittal (**a**, **b**, **c**), axial (**d**, **e**, **f**), T2-weighted (arrows on **a, d**), diffusion-weighted (arrows on **b, e**) images; subtle hyperintensity of sagittal (arrow on **c**) and axial apparent diffusion coefficients (arrow in **f**) were noted at levels T11–12. MRI, magnetic resonance imaging

**Fig. 2:**
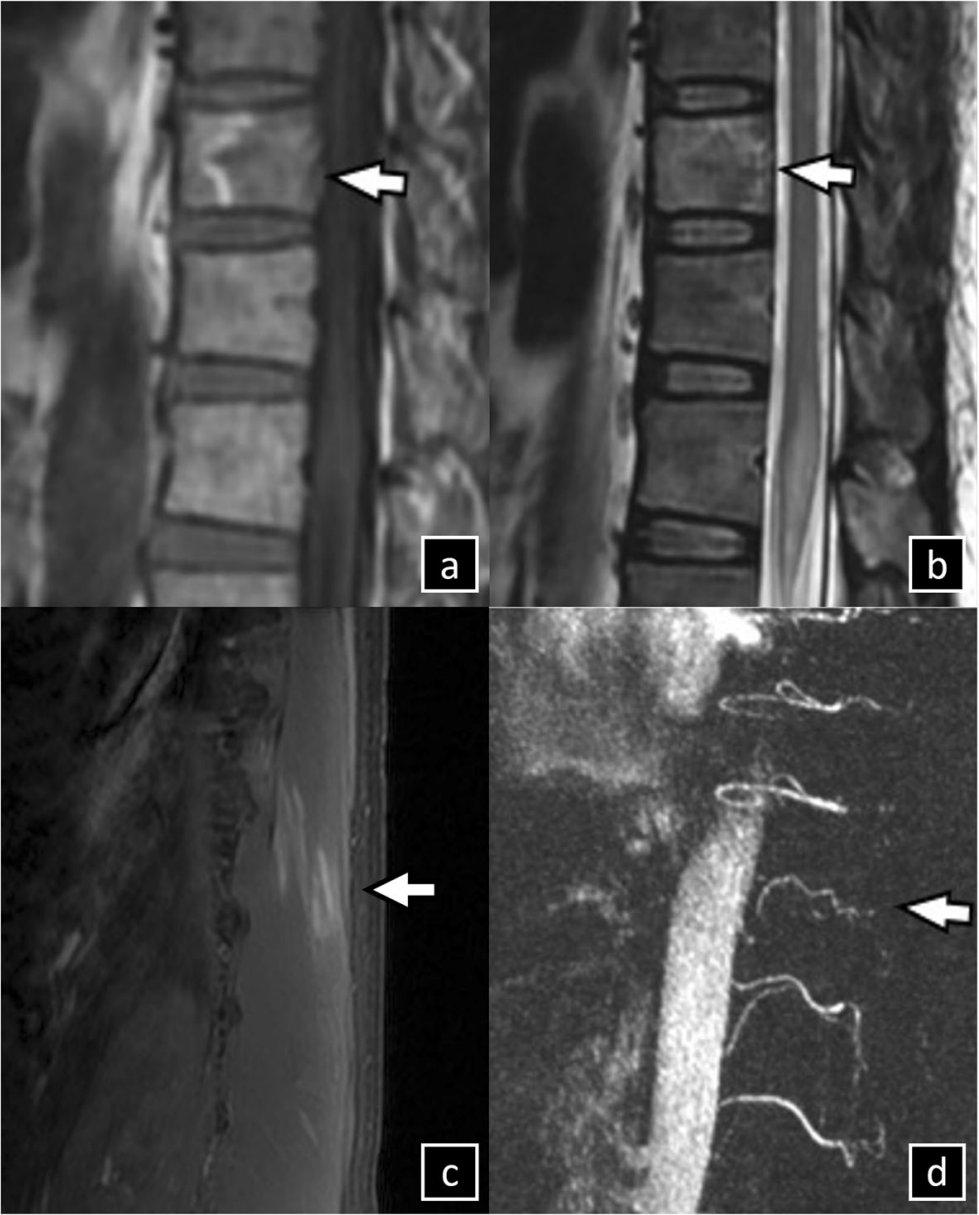
Sagittal view of spine MRI of patient 1. **a** High signal intensity on T1-weighted image and (**b**) low signal intensity on T2-weighted image at vertebral body T9 indicate vertebral bone infarction. **c** High signal intensity on apparent diffusion-coefficient image in erector spinae and (**d**) decreased blood flow in the supply artery on time-of-flight MRI at T10 also indicate muscle infarction. MRI, magnetic resonance imaging

Thoracolumbar MRI conducted 16 h after symptom onset revealed no cauda equina lesion but showed hyperintensity in T2-weighted and diffusion-weighted imaging (DWI) of the conus medullaris, confirming infarction (Fig. 1). Infarctions were also observed in the T9 vertebral body and T10 erector spinae muscles (Fig. 2). Initial nerve conduction and late response studies performed 12 days after symptoms onset showed decreased amplitudes of compound-muscle action potentials (CMAP) on left tibial nerves (right/left, 3.1/0.4 mV; reference, ≥3 mV), absence of H reflex response and F wave of the tibial nerve on the left side, and prolonged latency of the right H reflex response (32.4 ms; reference, < 31 ms). The needle EMG discloses no motor unit potentials on tentative contraction of the muscles gastrocnemius and left biceps femoris muscles, bilaterally. Serial nerve conduction studies revealed marked reduced amplitudes of CMAP in the left tibial nerve, and the needle EMG findings showed active denervation in bilateral S1 innervating muscles and chronic reinnervation on right S1 innervating muscles after 26 and 39 months. Additional file 1: Table S1 presents the complete findings of serial nerve conduction and needle EMG studies.

### Patient 2

A 34-year-old woman without hypertension, diabetes mellitus, dyslipidemia, smoking, migraine, oral contraceptive use, or other family history presented with acute onset of low back pain after sitting on the toilet. Over the next 7 h, she experienced progressing weakness and numbness in both lower limbs, with right side predominance. Urinary incontinence was noted 3 h after the onset of pain. The leg weakness progressed in a proximal to distal direction, reaching its peak after 7 h. The initial neurological examination conducted 16 h after symptom onset revealed asymmetric weakness in the lower limbs, decreased muscle strength in the distal right leg (score, 1 out of 5), right proximal leg (2 out of 5) and proximal and distal left leg (3 out of 5). Examination of sensory modalities revealed saddle dysesthesia, asymmetrically diminished pinprick sensation below dermatome T11, and impaired position sense in the left foot. Deep tendon reflexes were absent in both ankles and reduced in the right knee. As in patient 1, the symptoms were similar to those of cauda equina syndrome.

Laboratory tests conducted 3 days after symptom onset revealed no remarkable findings in complete blood count or in the concentrations of electrolytes, liver enzymes, blood urea nitrogen, creatinine, and low-density lipoprotein (85 mg/dL, normal range, < 130 mg/dL). Laboratory tests revealed mild elevations in D-dimer (533 FEU ng/mL; normal reference, ≤500 FEU ng/mL) and protein C (> 140%; normal range, 70–140%) but normal values for prothrombin time, partial thromboplastin time, erythrocyte sedimentation rate, and plasma levels of fibrinogen, protein S, serum antinuclear antibody, rheumatic factor, anti-ds DNA, anti-SSA/SSB antibodies, anticardiolipin immunoglobulin, and lupus anticoagulant. Human immunodeficiency virus antibody assay and Venereal Disease Research Laboratory tests were negative. CSF analysis demonstrated neither pleocytosis nor elevated protein levels on day 2 and 1 week later. The ECG results were normal. Transthoracic echocardiogram was unremarkable.

**Fig. 3:**
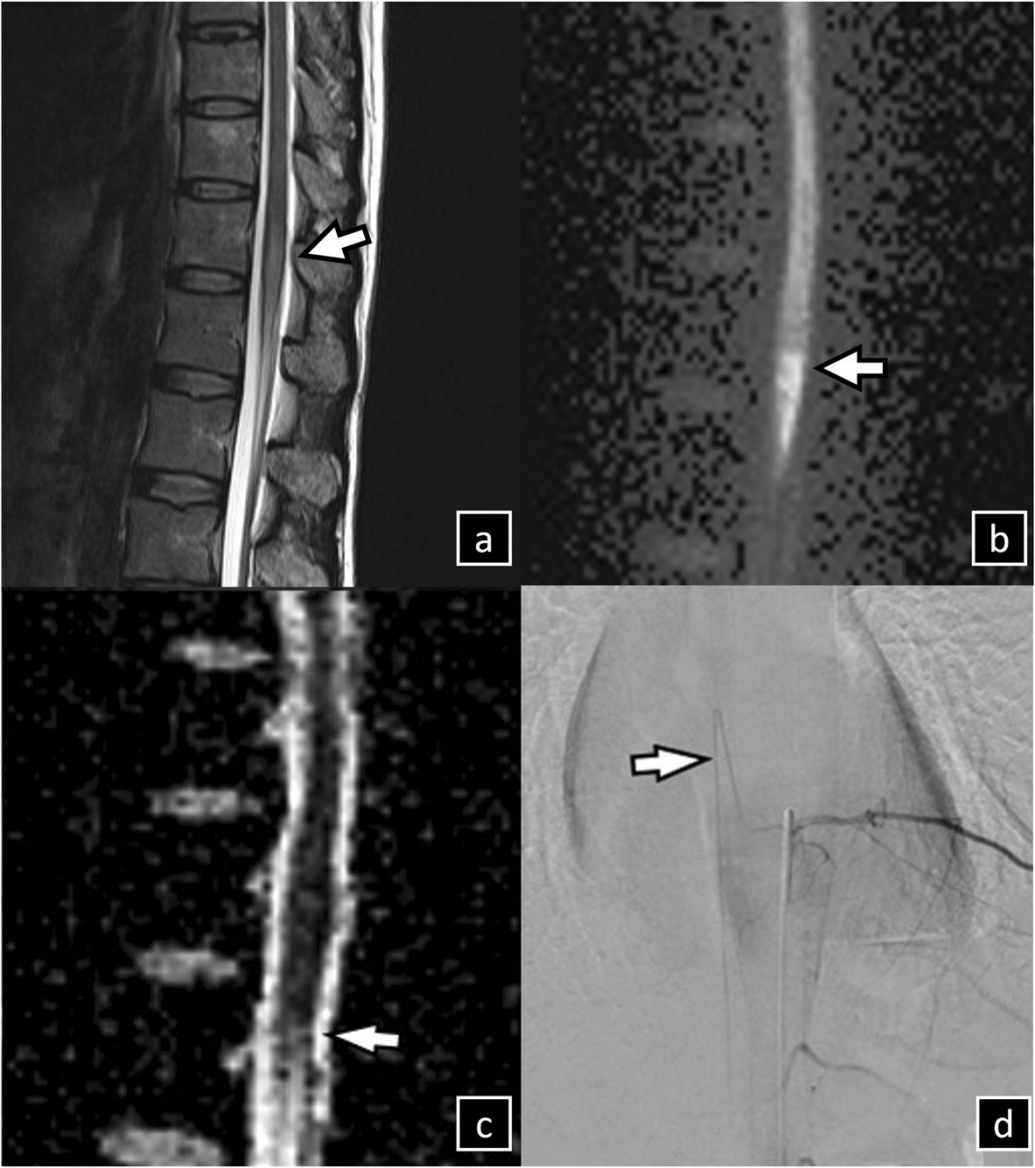
Spine images of patient 2. MRI, performed on day 2 following onset, shows typical hyperintensities on sagittal T2-weighted image (arrows in **a**) and diffusion-weighted image (arrows in **b**), as well as hypointensities on sagittal apparent diffusion coefficient image (arrows on **c**) in the conus medullaris T12. Spinal angiography revealed a patent anterior spinal artery, with the artery of Adamkiewicz arising at the left (**d**) T9 intercostal arteries. MRI, magnetic resonance imaging

Findings of the initial lumbar MRI, conducted 18 h after onset, were negative. Three days after the onset of symptoms, MRI of the spine revealed brightening in T2-weighted images and DWI in the conus medullaris but low signal intensity in the apparent diffusion coefficient image, confirming conus medullaris infarction instead of a cauda equina lesion. Spinal angiography revealed a patent anterior spinal artery, with the artery of Adamkiewicz arising from left T9 and T10 of the intercostal arteries (Fig. 3).

On day 1, nerve conduction study revealed a relatively low CMAP amplitude upon stimulation of the right tibial nerve at, above, and posterior to the medial malleolus (right/left, 4.2/15.2 mv); the absence of F wave on right deep peroneal nerve stimulation; and absence of both H reflex responses. No motor unit potentials were disclosed in the right L5-S1 myotome on needle EMG. The neurophysiological findings observed four months later supported severe right L5-S1 root lesion, moreover it was observed a progressive reduction of the right tibialis nerve CMAP amplitude and persistence of active denervation in the S1 territory bilaterally 8 to 51 months after onset (Additional file 1: Table S1).

The patient’s muscle strength improved gradually during hospitalization, and she could walk with aid after 3 weeks. Two months after discharge, she was able to move independently; however, mild urinary retention was observed. Four years later, she showed symptoms of asymmetric calf atrophy with right side predominance, absence of bilateral ankle jerk, and slight saddle dysesthesia with urine retention.

## Discussion and conclusions

Blood is supplied to the anterior two-thirds of the spinal cord by the anterior spinal artery, with the remainder supplied by the posterior spinal artery. The radiculomedullary artery, branching from the radicular artery from the aorta, feeds both the anterior and posterior spinal arteries, forming a collateral network of the arteria vasa corona. The Adamkiewicz artery is one of the thoracolumbar radicular arteries, a primary supplier of the lower thoracic and lumbar spinal cord, including the conus medullaris. The initial presentation of spinal cord infarction includes acute or sudden onset of back pain with bilateral sensory impairment. The onset of limb weakness as well as sphincter dysfunction varies and may be present at onset, reach a nadir within 12 h, or develop gradually [4]. Conus medullaris infarction is especially rare in SCI because of the relatively good blood supply to the conus medullaris from the Adamkiewicz artery and anastomosis of the anterior and posterior spinal arteries (anastomotic ansa) [5]. Conus medullaris infarction, a form of SCI, may be misdiagnosed as cauda equina syndrome before MRI study because of pain, sphincter dysfunction, reduced deep tendon reflex, and sometimes asymmetric neurological manifestations in the lower extremities [6–9]. The clinical features of conus medullaris infarction as presented in the literature are summarized in Table 1. Our 2 patients had clinical features similar to those described in the literature, underscoring the importance of recognizing these features for accurate diagnosis. When back pain and acute symptoms occur, conus medullaris infarction should be an important differential diagnosis.

**Table 1:** Summary of patient characteristics in published reports of infarct of the conus medullaris

Study	Patient Age (yrs/sex)	Cause of Infarct	Diseases, habits, medical history	Prognosis	NCV and EMG findings
Konno et al. [6]	77/F	NA	NA	NA	NA
Combarroso et al. [7]	69/F	NA	Hypertension	Walk with walker and normal in sphincter after 2 months	Absence of peroneal F waves from day 5 to month 9, spontaneous activity at anterior tibialis muscle from week 4 to month 12
Alanazy et al. [8]	48/M	Possible hyperextension posture	NA	Walk on walker on day 105	Absence of F waves on lower limbs on day 4 and slightly prolonged on day 56
Lamin et al. [10]	9/F	NA	NA	Ambulation independently	NA
Herrick et al. [11]	NA	Atheromatous emboli from aortic dissecting aneurysm	NA	NA	NA
Anderson et al. [12]	54/M	Aortic manipulation following MI post CABG	Heart failure, MI	Partial recovery and die of MI 7 weeks later	NA
	75/M	Aortic aneurysm operation	Smoking	Partial recovery after 16 months	NA
	66/M	Abdominal aorta calcification	Smoking	Partial recovery after 2 months	NA
	51/M		Smoking	Partial recovery after 28 months	NA
	47/F	NA	No	Stationary in 2 years	NA
Wildgruber et al. [13]	44/F	Prothrombin mutation	OCP use	Partial recovery in sphincter and ambulation after 2 weeks	NA
Mhiri et al. [14]	28/M	Dural arteriovenous fistula	NA	Stationary	NA
Diehn et al. [15]	24/M	Possible fibrocartilaginous emboli	NA	Stationary	NA
Wong et al. [16]	79/F	Diffuse aortic atheroma, microvascular injury following CABG	Post CABG history, renal insufficiency	Partially recovery at discharge	NA
Andrews et al. [17]	71/F	NA	No	Walk without assistance and neurogenic bladder after 2 months	Decreased recruitment in gluteus maximus, biceps femoris, gastrocnemius, anterior tibialis and anal sphincter
Herkes et al. [18]	53/F	NA	NA	Walk with assistance after 6 months	NA
Our study	55/M	Prolonged lateral lying	Dyslipidemia	Normal in ADL and asymmetric calf atrophy and neurogenic bladder in 39 months	Persistent active denervation in both S1 innervating muscles
	34/F	Prolonged sitting on a toilet	No	Normal in ADL, asymmetric calf atrophy and neurogenic bladder in 51 months	Persistent active denervation in both S1 innervating muscles

*NCV* Nerve conduction studies, *EMG* Electromyogram, *NA* Not available, *CABG* Coronary artery bypass graft, *OCP* Oral contraceptive pill, *ADL* Daily activity of living

The risk factors for SCI include cardiac emboli, aortic or vertebral artery disease, ischemic events during aorta surgery, degenerative disease of the spine, systemic hypotension, and atherosclerotic disease [3, 10, 19]. The etiology of conus medullaris infarction includes severe intervertebral disc herniation, atheromatous emboli from an aortic dissecting aneurysm, aorta calcification, hypo-perfusion, coagulopathy, and vascular abnormality [11–14, 20]. Abnormalities of the collateral vascular supply and the occlusion of feeding arteries are considered possible causes of spontaneous infarction of the conus medullaris [12]. Hyper-extension of the back has been reported as a cause of conus medullaris infarction [8]. In addition, special operation postures such as sitting, head flexion, or back hyper-extension have been discussed as possible risk factors for spinal cord infarction due to epidural venous congestion, increased venous pressure, or alteration of spinal blood flow [21–26]. No clear risk factor is evident in either of the 2 patients reported in this study; inappropriate or prolonged positioning is implicated.

We found that MRI was useful for diagnosing SCI. Accurate and early diagnosis was made based on the presence of hyperintensive lesions in T2-weighted and diffusion-weighted images. Infarction of adjacent vertebral bodies or muscles sharing the same arterial supply can also occur [6, 15], as was observed in both of our patients. Angiography can be used for vascular survey of the aorta, radicular arteries, and spinal arteries but is less sensitive for the definitive diagnosis of conus medullaris infarction. CSF analysis is suggested for patients with spontaneous onset of unknown cause, but mild elevation in CSF total protein is not exclusive to spinal cord infarction. A recent study proposed diagnostic criteria for SCI, also emphasizing the importance of time course and specific MRI findings [27]. Both of our patients fit the criteria for definite SCI.

Nerve conduction studies and electromyography in conus medullaris infarction has been reported rarely [7, 8]. In patients with SCI, spontaneous activity is sometimes observed in needle EMG studies of paraspinal muscles and lower limbs [28]. Both upper and lower motor neuron involvement in SCI can be determined via nerve conduction studies [29]. In conus medullaris infarction, absence of the F wave after infarction and its reappearance is regarded as a sign of clinical improvement [7, 8]. The EMG evidence shows that active denervation occurs after conus medullaris infarction and can persistent up to 12 months after infarct [7]. Both of our patients presented with bilateral anterior horn cell involvement of the low lumbar and first sacral regions, with active denervation occurring even at the 4-year follow-up. The electrophysiological data correlate with the observed calf muscle atrophy and absence of ankle jerk reflex, which are indicative of severe ischemic damage to S1 anterior horn cells. The early loss of late responses in the affected segments, abnormal spontaneous activity, and persistent loss of motor units in S1 innervated muscles are consistent with marked weakness and atrophy of the calf muscles.

The prognosis for conus medullaris infarction is relatively good compared to other forms of SCI. Several studies report that of the patients with spinal cord infarction, nearly half were still severely impaired 2 months later or at the time of discharge from the hospital [3, 19]. The prognosis for SCI may depend upon the initial motor impairment and the extent of bladder dysfunction and proprioceptive impairment [2, 3]. Older patients and females tend toward poorer outcomes [19, 30, 31]. Conus medullaris infarction most often involves the lower lumbar to first sacral area; consequently, the morbidity of ambulation is less than that associated with other types of spinal cord infarction.

The limitations of this study are sample size, lack of bulbocavernosus reflex and anal sphincter function investigation, and absence of re-assessment of imaging studies. More patients are needed to confirm the clinical presentation and their unique EMG findings.

In conclusion, the presented clinical and laboratory findings from 2 patients with conus medullaris infarction should be useful for its accurate diagnosis, even in the absence of known risk factors. Poor recovery of nerve conduction at the first sacral myotome and asymmetric calf atrophy may be characteristic features of conus medullaris infarction.

## Supplementary information

**Additional file 1: Table S1.** Nerve conduction studies in two patients with conus medullaris infarction.

## Data Availability

All data generated or analyzed during this study are included in their entirety in this published article. The first author can provide the original data if needed.

## Abbreviations

CMAP: Compound-muscle action potentials
CSF: Cerebrospinal fluid
DTR: Deep tendon reflex
DWI: Diffusion-weighted imaging
ECG: Electrocardiogram
EMG: Electromyography
MRI: Magnetic resonance imaging
SCI: Spinal cord infarction

## References

[1] Sandson TA, Friedman JH (1989). Spinal cord infarction. Report of 8 cases and review of the literature. Medicine (Baltimore).

[2] Rubin MN, Rabinstein AA (2013). Vascular diseases of the spinal cord. Neurol Clin.

[3] Masson C, Pruvo JP, Meder JF, Cordonnier C, Touzé E, De La Sayette V, Giroud M, Mas JL (2004). Leys D; study group on spinal cord infarction of the French neurovascular society. Spinal cord infarction: clinical and magnetic resonance imaging findings and short term outcome. J Neurol Neurosurg Psychiatry.

[4] Ebner FH, Roser F, Acioly MA, Schoeber W, Tatagiba M (2009). Intramedullary lesions of the conus medullaris: differential diagnosis and surgical management. Neurosurg Rev.

[5] Monteiro L, Leite I, Pinto JA, Stocker A (1992). Spontaneous thoracolumbar spinal cord infarction: report of six cases. Acta Neurol Scand.

[6] Konno T, Suwabe T, Kasahara S, Umeda Y, Oyake M, Fujita N (2015). A case of conus medullaris infarction expanding to the vertebral bodies, major psoas and erector spinae muscles. Rinsho Shinkeigaku.

[7] Combarroso O, Sánchez-Pernaute R, Orizaola P, Berciano J (1995). Absence of F-waves as an early electrodiagnostic finding in infarction of the conus medullaris. Muscle Nerve.

[8] Alanazy MH (2016). Conus medullaris stroke. Does F wave predict return of ambulation?. Neurosciences (Riyadh).

[9] Ohbu S, Ishimoto A, Honda M, Fukuda H, Hata Y, Tada S (1990). Infarction of the conus medullaris. Eur Neurol.

[10] Lamin S, Bhattacharya JJ (2003). Vascular anatomy of the spinal cord and cord ischemia. Pract Neurol.

[11] Herrick MK, Mills PE (1971). Infarction of spinal cord. Two cases of selective gray matter involvement secondary to asymptomatic aortic disease. Arch Neurol.

[12] Anderson NE, Willoughby EW (1987). Infarction of the conus medullaris. Ann Neurol.

[13] Wildgruber D, Kuntz R, Kermer P, Bartel J, Fetter M, Dichgans J (1999). Elsberg syndrome due to infarction of the conus medullaris associated with a prothrombin mutation. J Neurol.

[14] Mhiri C, Miladi MI, Triki C, Kechaou MS (2000). Sacral meningeal arteriovenous fistula supplied by branches of the hypogastric artery revealed by conus medullaris infarction. Spinal Cord.

[15] Diehn FE, Hunt CH, Lehman VT, Schwartz KM, Eckel LJ, Black DF (2015). Vertebral body infarct and ventral cauda equina enhancement: two confirmatory findings of acute spinal cord infarct. J Neuroimaging.

[16] Wong JJ, Dufton J, Mior SA (2012). Spontaneous conus medullaris infarction in a 79 year-old female with cardiovascular risk factors: a case report. J Can Chiropr Assoc.

[17] Andrews BT, Kwei U, Greco C, Miller RG (1991). Infarct of the conus medullaris simulating a spinal cord tumor: case report. Surg Neurol.

[18] Herkes GK, Selby G, Sorby WA (1989). Infarction of the conus medullaris--clinical and radiographic features. Clin Exp Neurol.

[19] Novy J, Carruzzo A, Maeder P, Bogousslavsky J (2006). Spinal cord ischemia: clinical and imaging patterns, pathogenesis, and outcomes in 27 patients. Arch Neurol.

[20] McKinley W, Santos K, Meade M, Brooke K (2007). Incidence and outcomes of spinal cord injury clinical syndromes. J Spinal Cord Med.

[21] Martínez-Lage JF, Almagro MJ, Izura V, Serrano C, Ruiz-Espejo AM, Sánchez-Del-Rincón I (2009). Cervical spinal cord infarction after posterior fossa surgery: a case-based update. Childs Nerv Syst.

[22] Nitta H, Yamashita J, Nomura M, Igarashi N (1997). Cervical spinal cord infarction after surgery for a pineal region choriocarcinoma in the sitting position: case report. Neurosurgery..

[23] Morandi X, Riffaud L, Amlashi SF, Brassier G (2004). Extensive spinal cord infarction after posterior fossa surgery in the sitting position: case report. Neurosurgery..

[24] Roberts DR, Roe J, Baudouin C (2003). Hyperlordosis as a possible factor in the development of spinal cord infarction. Br J Anaesth.

[25] Maduri R, Daniel RT, Diezi M, Cotting J, Messerer M (2017). Paraplegia after posterior fossa surgery in prone position: can we prevent it?. Childs Nerv Syst.

[26] Amoiridis GWJ, Langkafel M, Maiwurm D, Przuntek H (1996). Spinal cord infarction after surgery in a patient in the hyperlordotic position. Anesthesiology..

[27] Zalewski NL, Rabinstein AA, Krecke KN, Brown RD, Wijdicks EFM, Weinshenker BG (2019). Characteristics of spontaneous spinal cord infarction and proposed diagnostic criteria. JAMA Neurol.

[28] Levin KH, Daube JR (1984). Spinal cord infarction: another cause of "lumbosacral polyradiculopathy". Neurology..

[29] Little JW, Goldstein B, Gitter A, Haselkorn JK (1996). Spinal cord infarction: varying degrees of upper and lower motoneuron dysfunction. J Spinal Cord Med.

[30] Salvador de la Barrera S, Barca-Buyo A, Montoto-Marqués A, Ferreiro-velasco ME, Cidoncha-Dans M, Rodriguez-Sotillo A (2001). Spinal cord infarction: prognosis and recovery in a series of 36 patients. Spinal Cord.

[31] Nedeltchev K, Loher TJ, Stepper F, Arnold M, Schroth G, Mattle HP, Sturzenegger M (2004). Long-term outcome of acute spinal cord ischemia syndrome. Stroke..

