# Supplementary material for: Clinical, neuroimaging, and nerve conduction characteristics of spontaneous Conus Medullaris infarction
Source: BMC Neurol. 2019 Dec 17;19:328. doi: 10.1186/s12883-019-1566-1 (PMC6916224; doi:10.1186/s12883-019-1566-1)
Supplement: Supplementary file 1 — Additional file 1: Table S1. Nerve conduction studies in two patients with conus medullaris infarction. [file 12883_2019_1566_MOESM1_ESM.docx]

| **Table S1. Nerve conduction studies in two patients with conus medullaris infarction** | | | | | | | | | | | | | | | | | | | |
| --- | --- | --- | --- | --- | --- | --- | --- | --- | --- | --- | --- | --- | --- | --- | --- | --- | --- | --- | --- |
|  |  |  | Motor | | | | | | | | |  | Sensory | | |  |  |  |  |
| P | Time since onset |  | Fibular nerve | | | |  | Tibial nerve | | | |  | Sural nerve | | |  | H reflex (ms)(ms) |  | EMGEMG |
|  |  |  | DL  (ms) | Amp  (mV) | NCV  (m/s) | F wave  (ms) |  | DL  (ms) | Amp  (mV) | NCV  (m/s) | F wave  (ms) |  | DL  (ms) | Amp  (uV) | NCV  (m/s) |  |  |  |  |
| 1 | 12 days | R | 4.2 | 3.2 | 45 | 46.9 |  | 3.7 | 3.1† | 41 | 50.3 |  | 2.4 | 12.5 | 58 |  | 32.4† |  | no motor unit in both S1 innervating muscles |
|  |  | L | 4.0 | 3.0 | 46 | 46.9 |  | 3.4 | 0.4† | 45 | NR† |  | 2.7 | 12.2 | 52 |  | NR† |  |  |
|  | 3 months | R | 5.2† | 3.2 | 43 | 47.7 |  | 4.2 | 3.5† | 41 | 49.7 |  | 2.5 | 16.8 | 56 |  | 39† |  | active denervation of both S1 innervating muscles |
|  |  | L | 5.7† | 4.2 | 43 | 47.7 |  | 5.3 | 0.4† | 46 | NR† |  | 3.0 | 12.8 | 47 |  | 40.6† |  |  |
|  | 12 months | R | 4.4 | 3.8 | 49 | 49.1 |  | 3.4 | 4.6 | 43 | 48.6 |  | 2.5 | 11.4 | 56 |  | 35.0† |  | active denervation of bilateral S1 innervating muscles |
|  |  | L | 4.0 | 4.0 | 45 | 46.9 |  | 4.0 | 0.4† | 47 | 56.2† |  | 2.4 | 11.0 | 58 |  | NR† |  |  |
|  | 26 months | R | 5.1† | 4.3 |  | 49.0 |  | 4.5 | 6.6 |  | 48.6 |  |  |  |  |  | 33.2† |  | active denervation and reinnervation of left S1 innervating muscles |
|  |  | L | 4.1 | 5.2 | 45 | 49.0 |  | 5.7† | 0.6† | 42 | 58.3† |  | 3.0 | 9.1 | 47 |  | NR† |  |  |
|  | 39 months | R | 4.5 | 5.7 | 42 | 44.6 |  | 4.1 | 7.7 | 40 | 50.1 |  | 2.5 | 16.1 | 56 |  | 32.7† |  | active denervation of bilateral S1 innervating muscles with reinnervation on left side |
|  |  | L | 4.8 | 5.8 | 46 | 45.7 |  | 5.0 | 0.8 | 51 | 55.6† |  | 2.7 | 15.2 | 52 |  | NR† |  |  |
|  |  |  |  |  |  |  |  |  |  |  |  |  |  |  |  |  |  |  |  |
| 2 | 1 day | R | 5.0 | 4.0 | 40 | NR† |  | 6.1† | 4.2 | 41 | 50.5 |  | 2.9 | 25.2 | 47 |  | NR† |  | no motor unit in right L5 and S1 innervating muscles |
|  |  | L | 4.3 | 5.8 | 42 | 51.9† |  | 5.4 | 15.2 | 41 | 51.4† |  | 2.1 | 13.3 | 43 |  | NR† |  |  |
|  | 4 months | R | 5.5† | 1.2† | 46 | 47.1 |  | 6.0† | 0.1† | 50 | NR† |  | 2.7 | 30.9 | 52 |  | NR† |  | active denervation of right L5 and S1 innervating muscles |
|  |  | L | 3.7 | 2.3 | 43 | 41.1 |  | 4.1 | 7.1 | 47 | 35.4 |  | 2.9 | 7.7 | 48 |  | 33.0† |  |  |
|  | 8 months | R | 3.7 | 0.8† | 47 | 40.5 |  | 3.6 | 0.1† | 67 | NR† |  | 2.4 | 21.5 | 58 |  | 28.5 |  | active denervation of right L5 and both S1 innervating muscles |
|  |  | L | 3.8 | 1.6† | 54 | 40.5 |  | 3.4 | 7.8 | 49 | 44.5 |  | 2.0 | 12.4 | 70 |  | 28.8 |  |  |
|  | 14 months | R | 4.0 | 1.1† | 45 | 43.5 |  | 4.0 | 0.5† | 47 | 45.1 |  | 2.3 | 29.0 | 61 |  | 32.6† |  | active denervation of both S1 innervating muscles and reinnervation in right side |
|  |  | L | 4.5 | 2.1 | 48 | 41.6 |  | 3.6 | 11.9 | 49 | 44.5 |  | 2.2 | 11.2 | 64 |  | 31.7† |  |  |
|  | 26 months | R | 3.4 | 1.3† | 45 | 44.4 |  | 3.6 | 0.6† | 50 | 47.6 |  | 2,5 | 29.4 | 56 |  | 32.6† |  | active denervation and reinnervation of both S1 innervating muscles |
|  |  | L | 3.9 | 2.2 | 47 | 42.4 |  | 3.5 | 13.6 | 47 | 45.8 |  | 2.3 | 8.2† | 61 |  | 33.1† |  |  |
|  | 38 months | R | 4.0 | 1.4† | 44 | 43.5 |  | 3.5 | 0.6† | 44 | NR† |  | 2.5 | 39.4 | 56 |  | NR† |  | active denervation and reinnervation of both S1 innervating muscles |
|  |  | L | 4.5 | 2.6 | 46 | 43.5 |  | 3.5 | 15.0 | 45 | 48.8 |  | 2.3 | 14.6 | 61 |  | NR† |  |  |
|  | 51 months | R | 3.6 | 2.4 | 42 | 40.5 |  | 3.4 | 0.5† | 43 | NR† |  | 2.2 | 28.5 | 64 |  | NR† |  | active denervation of both S1 innervating muscles and reinnervation of left side |
|  |  | L | 3.7 | 2.7 | 47 | 41.2 |  | 3.2 | 12.9 | 47 | 44.6 |  | 2.0 | 13.8 | 70 |  | NR† |  |  |
|  | Normal mean (n=40) |  | 4.2 | 5.9 | 51.1 | <50 |  | 5.3 | 9.8 | 50.5 | <51 |  | 3.0 | 29.0 | 49.4 |  | <31 |  |  |
|  | SD |  | 0.5 | 2.5 | 3.2 |  |  | 1.0 | 2.9 | 3.4 |  |  | 0.3 | 15.1 | 4.2 |  |  |  |  |
| EMG, electromyogram; NCV, nerve conduction velocity; DL, distal latency; Amp, amplitude; L, left; R, right; NR, no response; SD, standard deviation; †, abnormal data, abnormal >3SDs beyond the mean or <2.0 mV amplitude in the peroneal motor nerves, <3.0 mV in the tibial motor nerves, and <10 μV in the median sensory and sural nerves | | | | | | | | | | | | | | | | | | | |
